# Efficacy and Safety of Aldosterone Synthase Inhibitors in Hypertension: A Systematic Review and Meta‐Analysis

**DOI:** 10.1002/edm2.70094

**Published:** 2025-09-12

**Authors:** Jia Shen Goh, Sameen Sohail, Haroon Ayub, Zian Zafar Cheema, Nitish Behary Paray, Sanka Adikari, Ahmad Mesmar, Mohammad Atout, Abdul Rehman Qazi, Ahmad Aldalqamouni, Bilal Younas, Muhammad Atif Rauf, Muhammad Azhar Waheed Khan, Aya Abouayana, Ahmed Eid Ahmed Abouayana, Ali Hasan, Maryam Shahzad, Mushood Ahmed, Raheel Ahmed, Saeed Ahmed

**Affiliations:** ^1^ Department of Renal Medicine South Tyneside and Sunderland NHS Foundation Trust Sunderland UK; ^2^ Department of Medicine North Tees and Hartlepool NHS Foundation Trust Stockton‐on‐Tees UK; ^3^ Department of Nephrology Indus Hospital, Lahore Pakistan; ^4^ Department of Nephrology Sialkot Medical College Lahore Pakistan; ^5^ Department of Medicine Amna Inayat Medical College Sheikhupura Pakistan; ^6^ Department of Cardiology Somerset NHS Foundation Trust Taunton UK; ^7^ Department of Acute Internal Medicine Ulster Hospital Belfast UK; ^8^ Department of Cardiology, Sheikh Shakhbout Medical City UAE; ^9^ Department of Internal Medicine The Specialty Hospital Amman Jordan; ^10^ Department of General Surgery South Tyneside District Hospital South Shields UK; ^11^ Department of Medicine The Specialty Hospital Amman Jordan; ^12^ Department of Respiratory Medicine South Tyneside and Sunderland NHS Foundation Trust Sunderland UK; ^13^ Department of Cardiology Armed Forces Institute of Cardiology (AFIC) Rawalpindi Pakistan; ^14^ Department of Nephrology University Hospital Monklands UK; ^15^ Department of Cardiology Sheikh Shakhbout Medical City UAE; ^16^ Department of Internal Medicine Saudi German Hospital UAE; ^17^ Department of Medicine Imperial College London UK; ^18^ Department of Medicine Dow University of Health Sciences Karachi Pakistan; ^19^ Department of Medicine Rawalpindi Medical University Rawalpindi Pakistan; ^20^ National Heart & Lung Institute, Imperial College London London UK; ^21^ Department of Cardiology Royal Brompton Hospital London UK; ^22^ Department of Nephrology Sunderland Royal Hospital UK

## Abstract

**Background:**

Hypertension remains a major contributor to global cardiovascular morbidity and mortality. Aldosterone, a key hormone in blood pressure regulation, plays a significant role in hypertension pathophysiology. This has led to growing interest in aldosterone synthase inhibitors (ASIs) as a potential treatment. This meta‐analysis aims to evaluate the efficacy and safety of ASIs in managing hypertension.

**Methods:**

A systematic search of PubMed, Google Scholar and Cochrane Central was conducted up to 13 July 2025, to identify randomised controlled trials (RCTs) evaluating ASIs in hypertensive adults. Data were analysed using RevMan version 5.4, employing random‐effects models with significance set at *p* < 0.05.

**Results:**

A total of 8 RCTs were included, with a total of 2003 participants in the ASI group and 650 participants in the placebo group. ASIs significantly reduced systolic blood pressure (SBP) compared to placebo (MD: −6.01 mmHg; 95% confidence interval [CI]: −9.31 to −2.71; I^2^ = 85%; *p* = 0.0004); diastolic blood pressure (DBP) was found to be comparable between the two groups (MD: −2.20 mmHg; 95% CI: −4.46 to 0.06; I^2^ = 69%; *p* = 0.06). There was a significant reduction in serum aldosterone levels favouring ASI use (MD: −1.46; 95% CI: −2.76 to −0.16; I^2^ = 99%; *p* < 0.00001). The risk of serious (RD: 0.00; 95% CI: −0.01 to 0.02; I^2^ = 30%; *p* = 0.75) and non‐serious adverse events (RD: 0.05; 95% CI: −0.02 to 0.12; I^2^ = 64%; *p* = 0.20) did not differ significantly between ASI and placebo groups. However, ASI use was associated with a significantly higher risk of hyperkalemia (RD: 0.04; 95% CI: 0.02 to 0.06; I^2^ = 70%; *p* = 0.002).

**Conclusion:**

ASIs effectively lower SBP and serum aldosterone in adults with hypertension. They appear safe overall but may increase the risk of hyperkalemia.

## Graphical abstract

ASIs significantly lowered systolic blood pressure (*P* = 0.0004) and serum aldosterone levels (*P* < 0.00001) compared to placebo across 8 RCTs. However, no significant difference was observed in diastolic blood pressure (*P* = 0.06) or in the risk of serious (*P* = 0.75) and non‐serious adverse events (*P* = 0.20). Notably, ASI use was associated with an increased risk of hyperkalemia (*P* = 0.002).

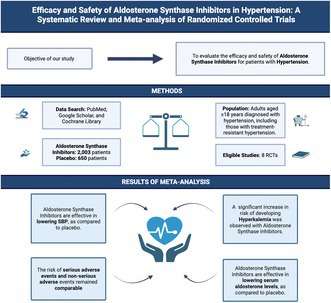

## 1 Introduction

Hypertension remains the leading contributor to cardiovascular morbidity and mortality worldwide. In the United States, a substantial proportion of individuals with hypertension continue to have inadequately controlled blood pressure (BP) [1, 2]. The increasing global prevalence of hypertension has paralleled rising obesity rates. In the United States, the age‐adjusted prevalence of hypertension—defined as systolic BP ≥ 130 mmHg, diastolic BP ≥ 80 mmHg or the use of antihypertensive medications—was 47.9% in 1999–2000, 43.0% in 2009–2010 and 44.7% in 2017–2020; affecting around 115.3 million U.S. adults during the 2017–2021 period [3].

Aldosterone plays a central role in BP regulation and the maintenance of sodium and potassium homeostasis and is implicated in the pathophysiology of cardiovascular and renal disease [4, 5, 6]. Elevated aldosterone levels have been associated with the onset of hypertension and are believed to play a significant role in mediating and exacerbating resistant hypertension; a condition affecting approximately 20% to 28% of individuals with hypertension [7, 8, 9]. While primary aldosteronism was previously thought to be rare (< 1% of unselected hypertensive patients), more recent evidence suggests a prevalence closer to 10%, with these individuals exhibiting greater cardiovascular damage even after adjusting for age, sex and BP levels [10].

Aldosterone synthase inhibitors (ASIs) represent a novel therapeutic approach by targeting the synthesis of aldosterone at its source, rather than merely antagonising its receptor [11, 12]. This mechanism addresses a key pathophysiological driver of hypertension and may offer improved efficacy and tolerability over existing therapies [11, 12].

Emerging data highlight the contribution of aldosterone excess to treatment‐resistant hypertension, particularly among individuals with obesity and comorbid conditions such as obstructive sleep apnea and metabolic syndrome [13, 14]. Current clinical guidelines recommend the addition of spironolactone, a mineralocorticoid receptor antagonist (MRA), as a fourth‐line therapy for resistant hypertension; however, its use is often limited by dose‐dependent adverse effects such as hypokalemia [15, 16].

Given the clinical relevance of aldosterone in hypertension pathogenesis and the potential role of ASIs as an emerging treatment strategy, we performed a systematic review and meta‐analysis to evaluate the efficacy, safety and overall therapeutic potential of ASIs in patients with hypertension.

## 2 Methods

### 2.1 Protocol Registration and Reporting Framework

This systematic review and meta‐analysis was conducted in accordance with the Preferred Reporting Items for Systematic Reviews and Meta‐Analyses (PRISMA) guidelines and methodological recommendations outlined in the Cochrane Handbook for Systematic Reviews of Interventions [17]. The protocol was prospectively registered in the PROSPERO international prospective register of systematic reviews under the identifier CRD420251059409, ensuring transparency and methodological integrity.

### 2.2 Data Sources and Search Strategy

A comprehensive and systematic literature search was developed and executed across MEDLINE (via PubMed), Google Scholar and the Cochrane Library from database inception through 13 July 2025, without restrictions on publication language. The search strategy was constructed using controlled vocabulary (e.g., MeSH terms) and relevant free‐text terms related to ‘aldosterone synthase inhibitors’, ‘blood pressure’, ‘hypertension’ and ‘randomized controlled trials’. The PICOS framework (Population, Intervention, Comparator, Outcomes, Study design) was utilised to define eligibility criteria and inform the structure of the search strategy. The full search strings for each database are included in the Table S1.

### 2.3 Data Extraction and Management

Two reviewers (M.A. and R.A.) independently performed data extraction using a piloted, standardised data collection form. The following variables were recorded: Participant demographics and baseline characteristics such as age, sex BMI, racial groups, duration of hypertension and ‘minimum background antihy‐ pertensive medications’ were extracted on an online Excel sheet. Similarly, a total of seven outcomes: ‘reduction in systolic blood pressure (SBP)’, ‘reduction diastolic blood pressure (DBP)’, ‘plasma aldosterone’, ‘serious adverse events’, ‘non‐serious adverse events’ and ‘hyperkalemia’ were extracted. Data were cross‐checked for accuracy by both reviewers, and any disagreements were resolved by consensus or adjudicated by a third reviewer when necessary (M.S.). Extracted data were managed using a secure, dedicated database.

### 2.4 Eligibility Criteria

This systematic review and meta‐analysis adhered to the PICOS (Population, Intervention, Comparator, Outcomes, Study design) framework to define inclusion and exclusion criteria [18].

#### 2.4.1 Population (P)

Adults aged ≥ 18 years diagnosed with hypertension, including those with treatment‐resistant hypertension.

#### 2.4.2 Intervention (I)

Oral administration of ASIs, such as Baxdrostat, Lorundrostat and Osilodrostat.

#### 2.4.3 Comparator (C)

Placebo or standard antihypertensive therapy.

#### 2.4.4 Outcomes (O)

Primary outcomes included changes in SBD and DBP from baseline to study endpoint. Secondary outcomes encompassed the incidence of serious and non‐serious adverse events, changes in biochemical markers (e.g., plasma aldosterone levels) and hyperkalemia.

#### 2.4.5 Study Design (S)

Randomised controlled trials (RCTs) assessing the efficacy and safety of ASIs in hypertensive patients.

### 2.5 Exclusion Criteria

Studies were excluded if they were not conducted on humans, were outside the scope of the current review or were published as review articles, editorials, letters, comments or conference proceedings. Case reports or small case series involving fewer than 10 patients were excluded, as were articles lacking sufficient information to reassess the outcomes of interest evaluated in this meta‐analysis. Additional exclusions included non‐randomised or observational study designs, studies involving paediatric populations (< 18 years) and those lacking a placebo or standard therapy comparator group. Studies were also excluded if they did not report data on blood pressure outcomes or adverse events, focused on secondary hypertension etiologies other than primary aldosteronism, were duplicate publications or had overlapping datasets. Reference lists of included studies and relevant meta‐analyses were screened to identify additional eligible studies. A list of all excluded studies has been included in Table S2.

### 2.6 Risk of Bias Assessment

We evaluated the methodological quality of included trials using the Cochrane Risk of Bias 2.0 (RoB 2) tool, which assesses bias across five domains [19]. Each domain was rated as having ‘low risk’, ‘some concerns’ or ‘high risk’ of bias. Two reviewers (M.A. and R.A.) independently conducted the assessments, with discrepancies resolved through discussion and consensus. Detailed results of the bias assessments are provided in Figure S1.

### 2.7 Statistical Analysis

All primary analyses were conducted using RevMan version 5.4. Forest plots were generated using a random‐effects model. For continuous outcomes, mean differences (MD) were reported, while risk differences (RD) were used for dichotomous outcomes along with the corresponding 95% confidence intervals (CIs). Statistical significance was defined as a *p*‐value less than 0.05.

Heterogeneity was assessed using Higgins' I^2^ statistic. An I^2^ value of 0%–50% was considered low, 50%–75% moderate and greater than 75% indicated high heterogeneity. When heterogeneity exceeded 75%, further investigation through sensitivity analysis was considered. Due to the inclusion of fewer than 10 RCTs, publication bias was not assessed, in accordance with recommended guidelines [20].

## 3 Results

### 3.1 Study Selection and Characteristics

**FIGURE 1:**
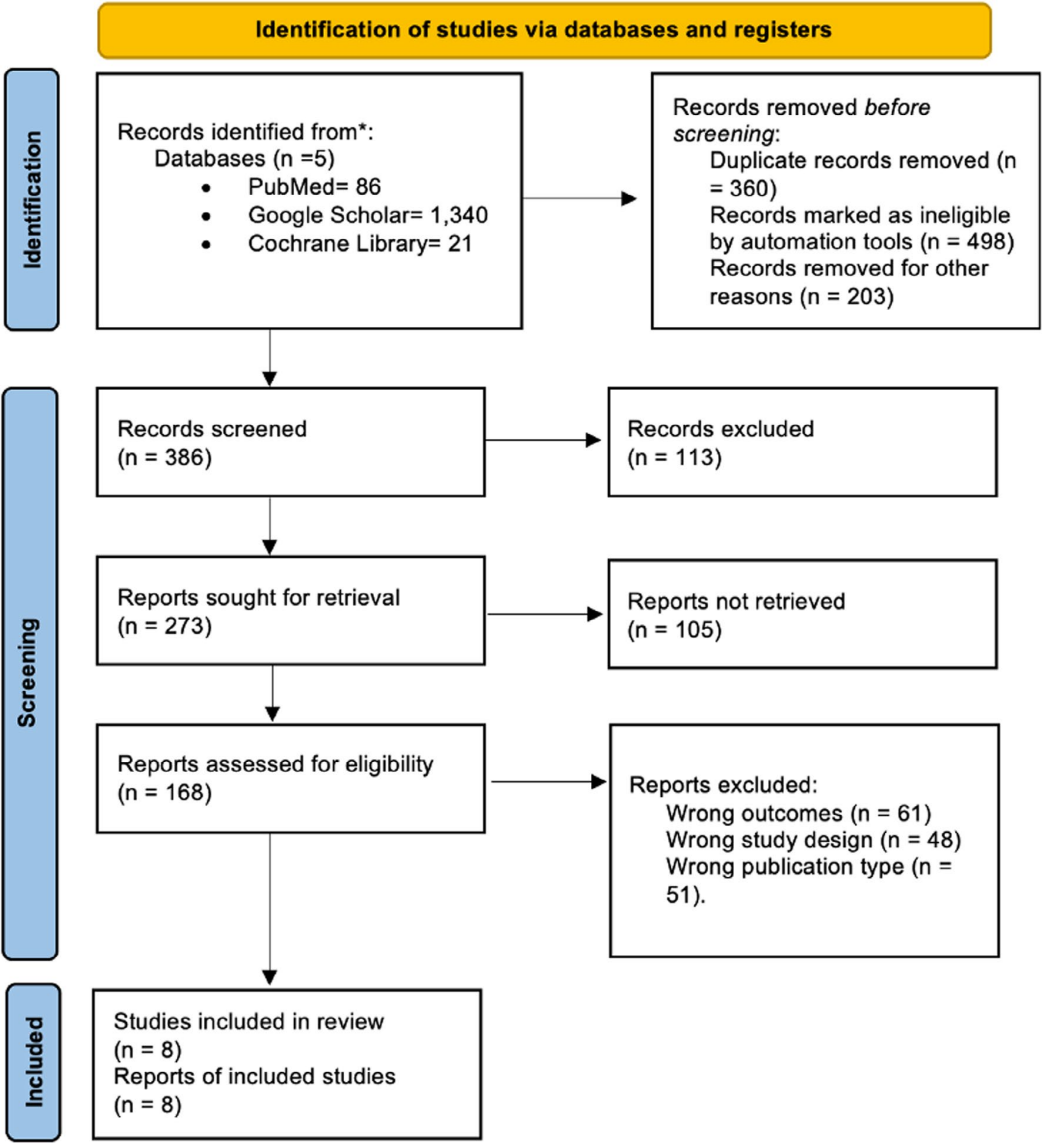
PRISMA chart depicting the study selection process.

Of the 1447 records initially identified through database searches, 1061 were removed prior to screening due to duplication, automation or other reasons. A total of 386 records were screened, of which 273 were sought for retrieval and 168 were assessed for eligibility. Following the exclusion of 161 reports due to reasons such as inappropriate outcomes, study design or publication type, eight studies met the inclusion criteria and were included in both the qualitative and quantitative synthesis [21, 22, 23, 24, 25, 26, 27, 28] (Figure 1). Of the eight included RCTs, two investigated Baxdrostat, three investigated Lorundrostat and three investigated Osilodrostat. The follow‐up duration across the included trials ranged from 8 to 12 weeks.

Participants in the ASI group were 2003, whereas the placebo group included 650 participants. The mean age of participants ranged from 53.6 to 68.7 years across the studies, with the proportion of men ranging from 48% to 70% depending on the trial. Ethnic distribution included predominantly White and Black/African American participants, with 433 Black participants (16.3%) and 1709 White participants (64.4%) overall. The mean BMI ranged from 30.4 to 34.5 kg/m^2^. Baseline SBP values ranged between 140.0 and 159.2 mmHg, while DBP ranged from 78.5 to 100.5 mmHg. Where reported, mean eGFR values ranged from 73.6 to 93.0 mL/min/1.73m^2^, indicating preserved renal function among most participants. Diabetes prevalence varied across trials, ranging from 6% to 46%, and heart failure status was reported in a minority of studies, with generally low incidence. The duration of hypertension, where available, ranged from 2 to 15.5 years. All participants were receiving at least one background antihypertensive medication, with many trials including patients on two or more agents (Table 1).

**TABLE 1:** Baseline characteristics of the included studies.

Study	Patients (*n*)	Drug	Age, mean (SD)	Male (*n*)	Black/African (*n*)	White (*n*)	BMI mean (SD)	Systolic BP (mean)	Diastolic BP (mean)	eGFR (mean)	Diabetes (*n*)	Heart failure (*n*)	Duration of HTN (years)	Min background antihypertensives
Mason W. Freeman	275	Baxdrostat 2 mg (*n* = 67)	61.2	38	19	47	33.5	147.3	88.2	85.2	31	—	—	≥ 1
		Baxdrostat 1 mg (*n* = 70)	62.7	37	20	48	31.9	147.7	87.7	83.2	20	—	—	
		Baxdrostat 0.5 mg (*n* = 69)	61.5	36	22	45	33.2	147.6	87.6	81	26	—	—	
		Placebo (*n* = 69)	63.8	42	16	51	32.1	148.9	88.2	85.5	28	—	—	
HALO trial	231	Baxdrostat 2 mg (*n* = 55)	59.2	30	14	44	—	146.3	—	—	—	—	—	≥ 1
		Baxdrostat 1 mg (*n* = 58)	61.2	25	15	43	—	147	—	—	—	—	—	
		Baxdrostat 0.5 mg (*n* = 57)	59.9	35	14	48	—	146.3	—	—	—	—	—	
		Placebo (*n* = 61)	60.5	27	17	46	—	147.9	—	—	—	—	—	
Luke J. Laffin	156	Lorundrostat 100 mg QD (*n* = 25)	68.7	12	15	14	30.4	142.2	78.5	77.4	8	1	—	≥ 2
		Lorundrostat 50 mg QD (*n* = 28)	64.7	13	8	19	32	140	84.7	77.2	8	1	—	
		Lorundrostat 25 mg BID (*n* = 28)	64.8	11	7	23	30.6	142.8	80.1	80.9	11	1	—	
		Lorundrostat 12.5 mg BID (*n* = 22)	68.1	8	7	15	32	142.6	81.6	81.7	9	1	—	
		Lorundrostat 12.5 mg QD (*n* = 23)	65.2	11	11	11	30.6	142.9	80.3	77.9	11	1	—	
		Placebo (*n* = 30)	62.6	13	16	13	31.9	142.9	83.8	81.6	14	0	—	
Adam D. Karns	122	Osilodrostat 0.25 mg BID (*n* = 32)	53.6	20	11	—	33.95	152.4	91.8	83.5	7	—	14.1	≥ 3
		Osilodrostat 1.0 mg QD (*n* = 26)	55.4	18	9	—	32.18	152.5	89.2	83.6	7	—	11.7	
		Osilodrostat 0.5 mg/1 mg BD (*n* = 31)	57.2	18	10	—	33.49	152.2	88.9	79.7	8	—	13.9	
		Placebo (*n* = 33)	59.8	22	11	—	31.92	153.4	90.1	76.9	6	—	15.5	
Karl Anderson	63	Osilodrostat 0.5 mg QD (*n* = 12)	56.1	8	—	—	31.19	15.1	87.8	89.5	2	—	10.9	≥ 1
		Osilodrostat 1.0 mg QD (*n* = 12)	54.2	7	—	—	33.63	14.9	90.4	89.1	4	—	11.7	
		Osilodrostat 1.0 mg BID (*n* = 13)	57.9	10	—	—	32.73	145.2	88.9	81.8	4	—	7.8	
		Osilodrostat 2.0 mg QD (*n* = 13)	56.2	8	—	—	30.7	146.2	85.6	78.4	1	—	10.2	
		Placebo (*n* = 13)	56.8	9	—	—	34.45	140.2	86.8	85.8	3	—	11.4	
David A. Calhoun	438	Osilodrostat 0.25 mg QD (*n* = 92)	53.9	63	5	86	—	157.7	100.4	86.3	—	—	6.3	≤ 2
		Osilodrostat 0.5 mg QD (*n* = 87)	54.8	58	11	76	—	157	99.9	84.5	—	—	6.7	
		Osilodrostat 1.0 mg QD (*n* = 86)	54.5	55	8	77	—	159.2	100	82.1	—	—	7.3	
		Osilodrostat 0.5 mg BID (*n* = 96)	54.4	63	10	85	—	158.5	100.2	86.5	—	—	6.2	
		Placebo (*n* = 77)	53.9	46	5	70	—	156.7	100.5	85.4	—	—	5.3	
ADVANCE‐HTN	285	Lorundrostat Stable Dose (*N* = 94)	61.3 ± 9.6	56	50	39	31.2	141.8	84.3	76.6	39	—	—	2–5
		Lorundrostat Dose Adjustment (*N* = 96)	60.9 ± 10.2	54	56	35	32.4	143.5	85.6	76.4	46	—	—	
		Placebo (*N* = 95)	59.1 ± 10.5	62	44	41	32.2	141.7	85.5	73.6	34	—	—	
LAUNCH‐HTN	1083	Lorundrostat 50 mg and then 100 mg (*N* = 270)	61.4 (10.3)	142	0	190	32.8	146.6	86.3	92.8	76	—	—	≥ 2
		Lorundrostat 50 mg (*N* = 541)	61.7 (10.6)	294	1	366	33	149	87.8	90.1	173	—	—	
		Placebo (*N* = 272)	61.8 (10.4)	139	1	177	32.6	148.8	87.1	91.2	89	—	—	

### 3.2 Outcomes

#### 3.2.1 Reduction in SBP

**FIGURE 2:**
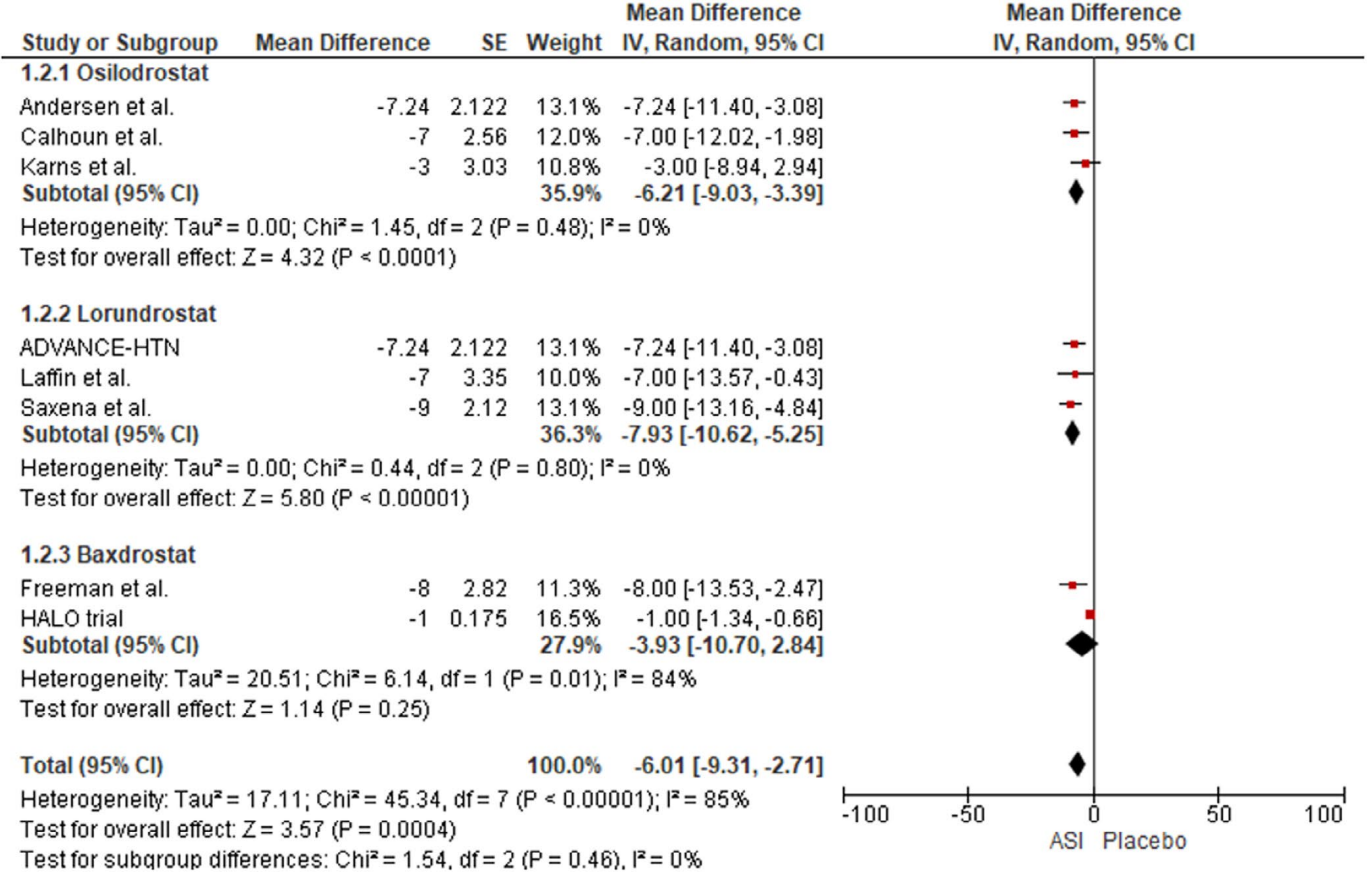
Forest plot for the outcome of Reduction in SBP.

Our analysis demonstrated a statistically significant reduction in SBP among patients treated with ASIs compared to placebo across the eight included studies (mean difference [MD]: −6.01 mmHg; 95% confidence interval [CI]: −9.31 to −2.71; I^2^ = 85%; *p* = 0.0004). In subgroup analysis, Osilodrostat (three studies) was associated with a significant SBP reduction (MD: −6.21 mmHg; 95% CI: −9.03 to −3.39; I^2^ = 0%; *p* < 0.0001). Similarly, pooled data from three trials evaluating Lorundrostat showed a substantial decrease in SBP (MD: −7.93 mmHg; 95% CI: −10.62 to −5.25; I^2^ = 0%; *p* < 0.00001). In contrast, no significant SBP reduction was observed in the Baxdrostat subgroup (MD: −3.93 mmHg; 95% CI: −10.70 to 2.84; I^2^ = 84%; *p* = 0.25) (Figure 2). A leave‐one‐out sensitivity analysis revealed that exclusion of the HALO trial from the Baxdrostat subgroup reduced heterogeneity from 85% to 0%, with a revised pooled effect size indicating a significant reduction in SBP (MD: −7.21 mmHg; 95% CI: −9.04 to −5.38; I^2^ = 0%; *p* < 0.00001) [24] (Figure S2).

#### 3.2.2 Reduction in DBP

**FIGURE 3:**
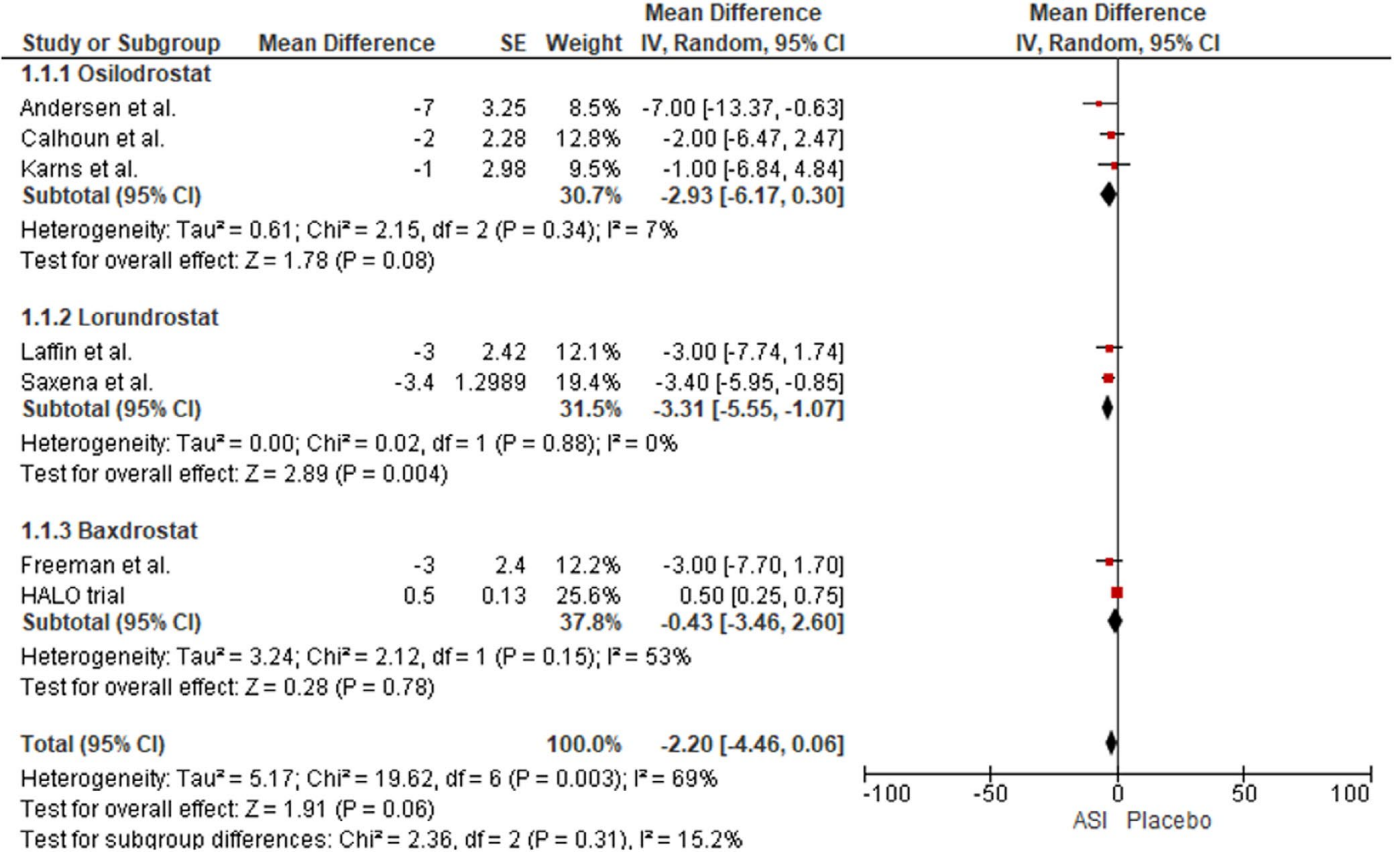
Forest plot for the outcome of Reduction in DBP.

Our analysis demonstrated no statistically significant change in DBP among patients treated with ASIs compared to those receiving placebo, based on seven included studies (MD: −2.20 mmHg; 95% CI: −4.46 to 0.06; I^2^ = 69%; *p* = 0.06). In the Osilodrostat subgroup, which included three studies, no significant change in DBP was observed (MD: −2.93 mmHg; 95% CI: −6.17 to −0.30; I^2^ = 7%; *p* = 0.08). Likewise, in the Baxdrostat subgroup, no significant difference in DBP was detected across the two pooled studies (MD: −0.43 mmHg; 95% CI: −3.46 to 2.60; I^2^ = 53%; *p* = 0.78). However, pooled results for Lorundrostat demonstrated a notable reduction in DBP (MD: −3.31 mmHg; 95% CI: −5.56 to −1.07; I^2^ = 0%; *p* = 0.004) (Figure 3). A leave‐one‐out sensitivity analysis demonstrated that removal of the HALO trial from the Baxdrostat subgroup reduced heterogeneity from 96% to 0%, yielding a significant DBP reduction (MD: −3.15 mmHg; 95% CI: −4.84 to −1.45; I^2^ = 0%; *p* = 0.0003) [24] (Figure S3).

#### 3.2.3 Serious Adverse Events

**FIGURE 4:**
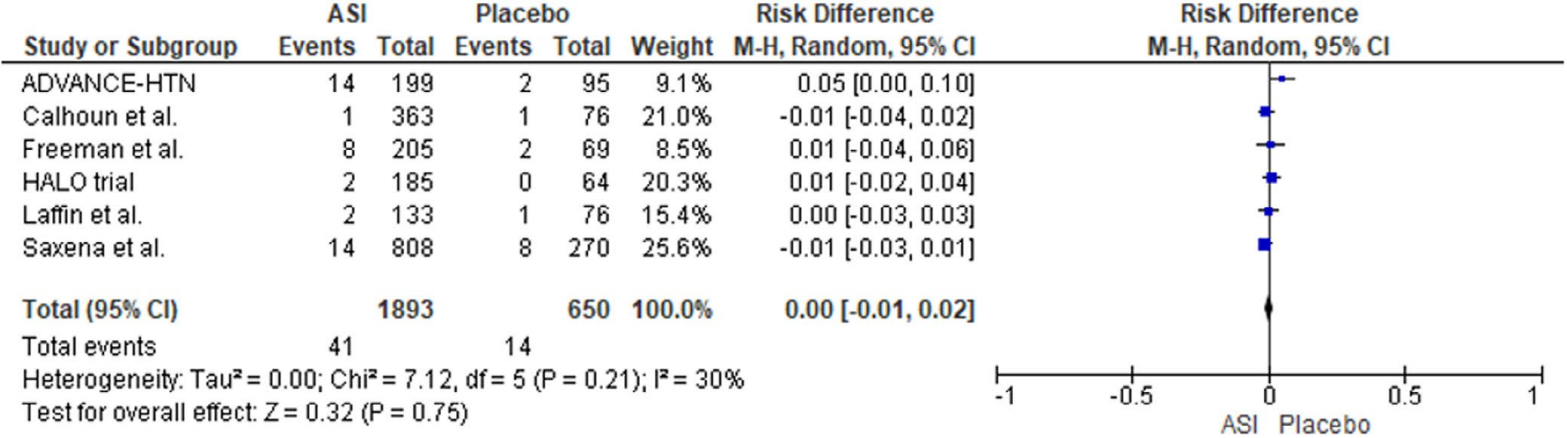
Forest plot for the outcome of any serious adverse event.

For the outcome of serious AEs, involving a total of 1 893 participants in the ASI group and 650 participants in the placebo group, the risk was found to be comparable between the two groups. The pooled analysis showed no statistically significant difference in the incidence of serious AEs (RD: 0.00; 95% CI: −0.01 to 0.02; I^2^ = 30%; *p* = 0.75), indicating that ASI use did not substantially increase the risk of serious adverse events compared to placebo (Figure 4).

We also conducted a leave‐one‐out analysis by removing the HALO trial, which caused the I2 to increase from 30% to 39% (RD: 0.00; 95% CI: −0.02 to 0.02; *p* = 0.88) [24] (Figure S4).

#### 3.2.4 Non‐Serious Adverse Events

**FIGURE 5:**
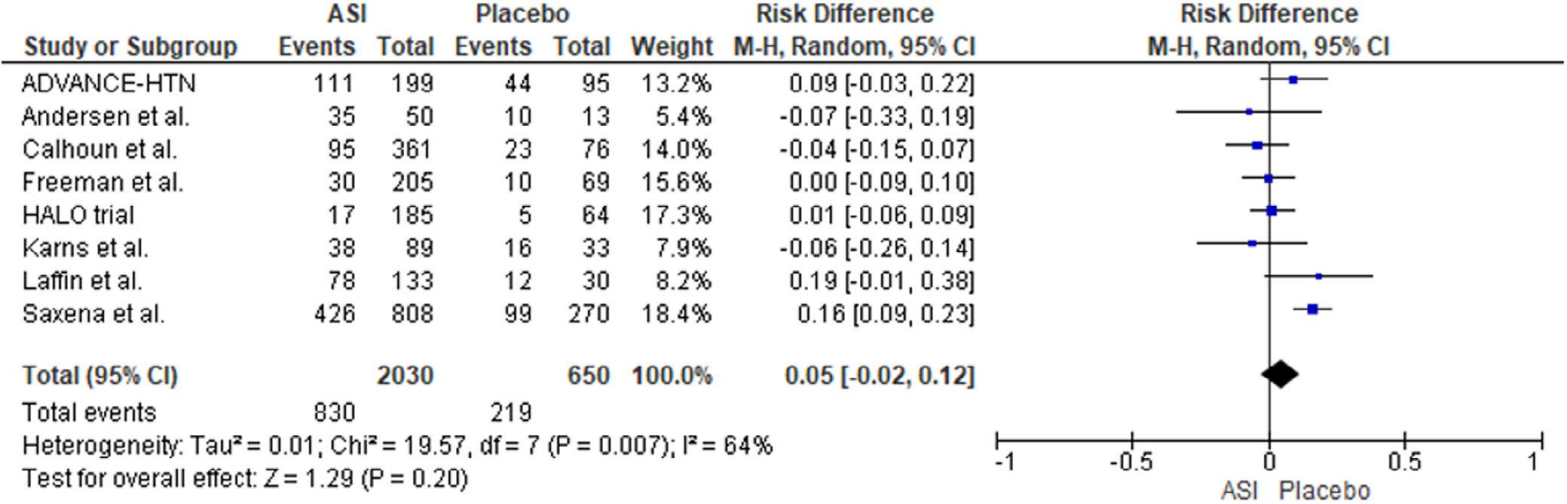
Forest plot for the outcome of non‐serious adverse event.

For the outcome of non‐serious AEs, involving a total of 2030 participants in the ASI group and 650 participants in the placebo group, the risk was found to be comparable between the two groups. The pooled analysis showed no statistically significant difference in the incidence of non‐serious AEs (RD: 0.05; 95% CI: −0.02 to 0.12; I^2^ = 64%; *p* = 0.20), indicating that ASI use did not substantially increase the risk of non‐serious AEs compared to placebo (Figure 5). A leave‐one‐out sensitivity analysis demonstrated that removal of the LAUNCH‐HTN trial reduced heterogeneity from 64% to 9%; however, the risk of non‐serious AEs remained comparable between the two groups (RD: 0.02; 95% CI: −0.03 to 0.07; I^2^ = 0%; *p* = 0.48) [28] (Figure S5).

We also conducted a leave‐one‐out analysis by removing the HALO trial, which caused the I^2^ to increase from 64% to 35% (RD: 0.05; 95% CI: −0.03 to 0.13; *p* = 0.22) [24] (Figure S6).

#### 3.2.5 Changes in Serum Aldosterone Level

**FIGURE 6:**
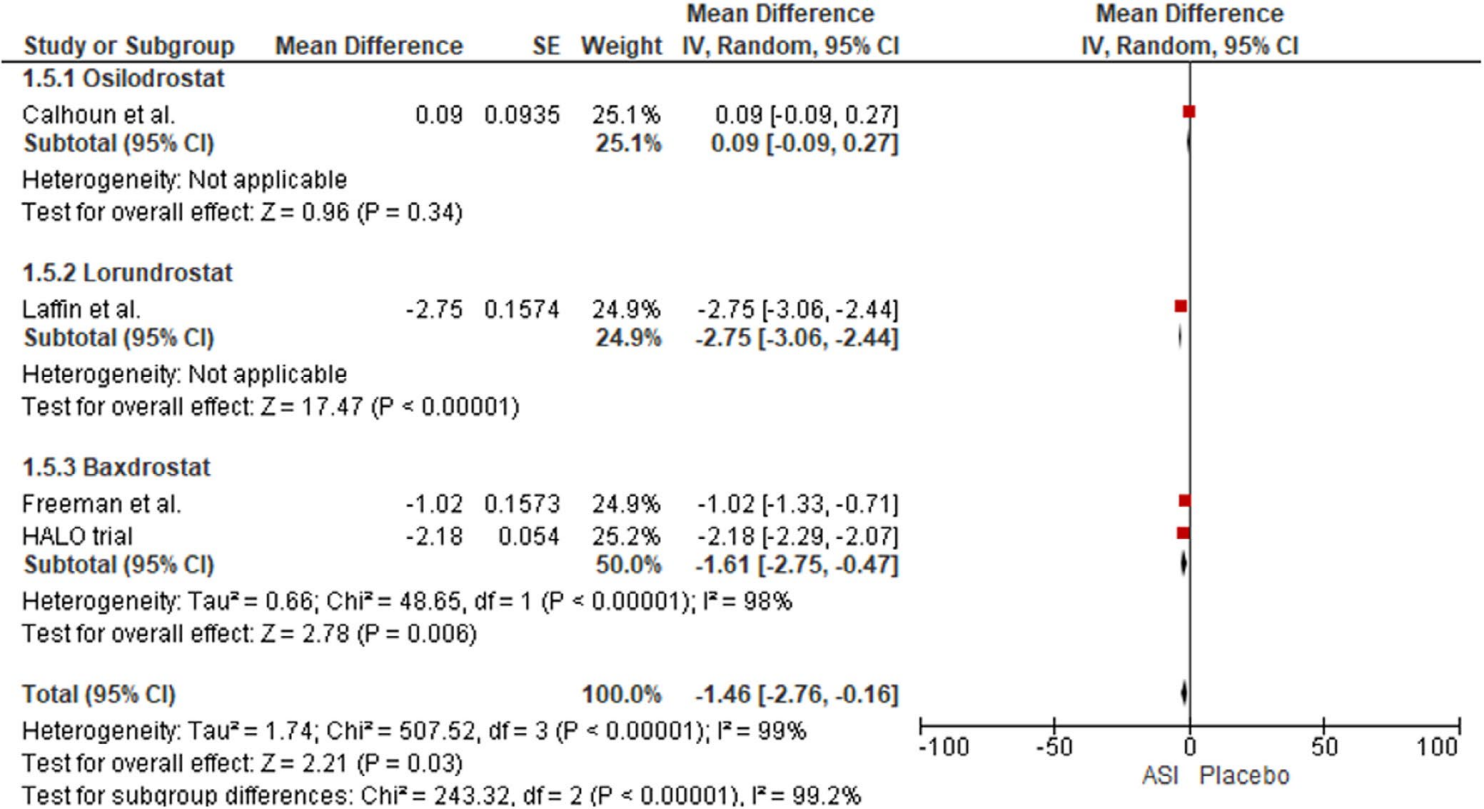
Forest plot for the outcome of changes in serum aldosterone.

Our analysis revealed a statistically significant reduction in serum aldosterone levels among patients treated with ASIs compared to those receiving placebo, based on data from 3 studies (MD: −1.46; 95% CI: −2.76 to −0.16; I^2^ = 99%; *p* < 0.00001) (Figure 6). In the Baxdrostat subgroup, a significant decrease in serum aldosterone levels was detected across the two pooled studies (MD: −1.61 mmHg; 95% CI: −2.75 to −0.47; I^2^ = 98%; *p* = 0.006). A leave‐one‐out sensitivity analysis demonstrated that removal of Calhoun et al. from the osilodrostat subgroup reduced heterogeneity from 99% to 97%, yielding a significant reduction in serum aldosterone levels (MD: −1.99; 95% CI: −2.77 to −1.20; I^2^ = 0%; *p* < 0.00001) [27] (Figure S7).

We also conducted a leave‐one‐out analysis by removing the HALO trial; the I^2^ remained unchanged; however, the change in serum aldosterone levels became comparable between the two groups (MD: 1.22; 95% CI: −2.90 to 0.46; *p* = 0.15) [24] (Figure S8).

#### 3.2.6 Risk of Hyperkalemia

**FIGURE 7:**
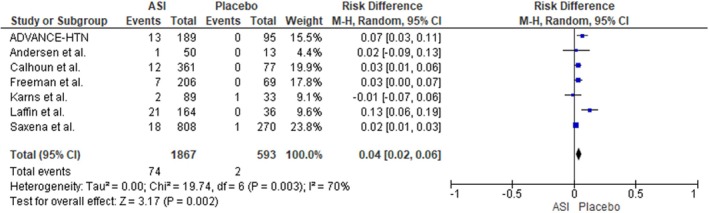
Forest plot for the outcome of Hyperkalemia.

For the outcome assessing the risk of hyperkalemia, data from a total of 1867 participants in the ASI group and 593 participants in the placebo group indicated a significantly elevated risk among patients receiving ASIs (RD: 0.04; 95% CI: 0.02 to 0.06; I^2^ = 70%; *p* = 0.002) (Figure 7). A leave‐one‐out sensitivity analysis demonstrated that removal of the RCT by Laffin et al. reduced heterogeneity from 70% to 38%, yielding a significant increase in the risk of developing hyperkalemia levels (RD: 0.03; 95% CI: 0.01 to 0.05; I^2^ = 38%; *p* = 0.0005) [23] (Figure S9).

## 4 Discussion

This systematic review and meta‐analysis assessed the efficacy and safety of ASIs in managing HTN, encompassing 1 084 participants in the ASI group and 380 in the placebo group. ASI treatment led to a statistically significant reduction in SBP and serum aldosterone levels; however, no changes were observed in DBP compared to placebo. Overall, ASIs were well tolerated, with similar rates of serious AEs and non‐serious AEs between groups, supporting a favourable safety profile. However, a notable increase in the risk of hyperkalemia was observed with ASIs, highlighting the need for careful patient monitoring to optimise outcomes.

Our results align with those of Marzano et al., who reported reductions in SBP and a comparable profile of adverse events, including an increased risk of hyperkalemia associated with ASIs. Unlike our analysis, Marzano et al. also observed a statistically significant decrease in DBP. Notably, their review included a broader set of trials involving patients with **chronic kidney disease, diabetes** and **albuminuric CKD**, whereas our study focused primarily on individuals with hypertension [29].

First, regarding changes in SBP, patients treated with ASIs showed a significant reduction compared to placebo. Subgroup analysis revealed that Osilodrostat led to a significant decrease, Lorundrostat showed the greatest reduction; whereas Baxdrostat failed to produce a modest reduction in SBP. These findings align with previous studies, including the ADVANCE‐HTN trial, where Lorundrostat significantly reduced 24‐h ambulatory SBP at week 12 in both the stable dose (*p* = 0.001) and dose‐adjustment groups (*p* = 0.006) [21]. Similarly, Andersen et al. found that while all four Osilodrostat (LCI699) dose groups lowered mean sitting SBP (MSSBP), only the two highest doses: 1.0 mg twice daily and 2.0 mg once daily, achieved statistically significant reductions (−12.5 mm Hg, *p* = 0.022 and −10.9 mm Hg, *p* = 0.046) [26].

ASIs primarily lower SBP by inhibiting aldosterone synthase (CYP11B2), thereby reducing aldosterone production, sodium retention, intravascular volume and vascular tone. Among ASIs, osilodrostat, though the first developed, lacked high selectivity and blunted ACTH‐stimulated cortisol in some patients, leading to its use in Cushing's disease [11, 30]. In contrast, lorundrostat is a highly selective ASI with a favourable safety profile. In a first‐in‐human study by Shimizu et al., involving single doses from 5 to 800 mg and multiple doses from 40 to 360 mg once daily, lorundrostat demonstrated predictable pharmacokinetics with peak plasma concentrations occurring within 1–3 h and a half‐life of 10–12 h [31]. It suppressed plasma aldosterone levels by up to 40% at doses of 100–200 mg and up to 70% at 400–800 mg, with levels returning to baseline within 16 h [31]. With regard to Baxdrostat, no significant change in SBP was observed. In the phase 2 BrigHTN trial, Baxdrostat at a dose of 2 mg daily led to a statistically significant placebo‐corrected reduction in seated systolic blood pressure of approximately 11.0 mmHg (*p* < 0.001) over 12 weeks in patients with treatment‐resistant hypertension [23]. In comparison, the HALO trial, which included patients with uncontrolled hypertension taking one or two background antihypertensives and had a shorter follow‐up of 8 weeks, showed reductions in SBP ranging from 16.0 to 19.8 mmHg in the Baxdrostat groups. However, the placebo group also experienced a similar reduction of 16.6 mmHg, resulting in no significant difference between groups [23, 24]. This finding is consistent with the high placebo response seen in the SYMPLICITY trials and may be explained by a Hawthorne effect, where participants demonstrated better adherence to their prescribed medications during the study. Additionally, white‐coat hypertension in some participants may have inflated baseline office BP values, contributing to the reduced apparent effect of the treatment [32, 33, 34].

Second, our analysis showed no significant reduction in DBP among patients receiving ASIs compared to placebo. Subgroup analyses revealed a notable DBP decrease with Lorundrostat; however, no significant change in DBP was observed for oslidrostat or baxdrosat. With regard to Lorundrostat, these findings are consistent with previous studies. Laffin et al. demonstrated that Lorundrostat 50 mg once daily significantly lowered diastolic automated office BP by 5.5 mm Hg (*p* = 0.02) [22]. In contrast, although Baxdrostat 2 mg reduced DBP by 14.3 ± 1.31 mm Hg, the between‐group difference (−5.2 mm Hg; 95% CI, −8.7 to −1.6) was not statistically significant in the BrigHTN trial [23]. As opposed to our results, Andersen et al. reported that only the 1.0 mg once‐ and twice‐daily Osilodrostat groups showed significant reductions in mean sitting DBP (−9.1 and −9.2 mm Hg; *p* = 0.009 and 0.007) [26].

These DBP‐lowering effects align with ASI's mechanism of inhibiting aldosterone synthase (CYP11B2), thereby reducing aldosterone, sodium retention and vascular resistance [11, 12, 30]. Variation in response may reflect pharmacologic differences; Lorundrostat's high selectivity for CYP11B2 may enhance efficacy, while Baxdrostat's lower observed effect could relate to its pharmacokinetics and partial nonadherence—36% of patients in HALO's 2 mg group had plasma levels < 0.2 ng/mL despite >95% adherence by pill count [24]. The lack of DBP effect in BrigHTN and HALO may be due to differences in trial populations and design [23, 24]. BrigHTN enrolled patients with treatment‐resistant hypertension, while HALO focused on those with uncontrolled hypertension. Additionally, the shorter treatment duration in HALO (8 weeks) compared to BrigHTN (12 weeks) may have limited the ability to detect the full antihypertensive effect [23, 24]. The limited effect of osilodrostat on DBP observed in our analysis can be explained by its off‐target pharmacological activity [35]. Osilodrostat inhibits CYP11B1, an enzyme crucial for cortisol synthesis [35, 36, 37]. This inhibition leads to an increase in adrenocorticotropic hormone (ACTH), which stimulates the adrenal cortex to produce higher levels of 11‐deoxycorticosterone (11‐DOC); a steroid precursor with mineralocorticoid receptor (MR) activity [35, 36]. The elevated 11‐DOC can counteract the antihypertensive effects expected from reduced aldosterone synthesis. Consequently, this unintended rise in MR‐active steroids likely blunted osilodrostat's overall blood pressure‐lowering impact [36]. In a trial by Schumacher et al., osilodrostat 1.0 mg/day showed a smaller reduction in systolic BP (−13.1 mmHg) compared to eplerenone 50 mg twice daily (−18.7 mmHg) in patients with resistant hypertension [36]. Similarly, a phase II trial evaluating osilodrostat in primary hypertension demonstrated that only the 1.0 mg/day dose resulted in a statistically significant DBP reduction (−7.1 mmHg), while lower doses did not differ from placebo [27]. These findings support the notion that osilodrostat's off‐target effects and dose‐dependent efficacy may underlie its inconsistent impact on DBP.

Third, regarding changes in serum aldosterone levels, our analysis revealed a significant reduction in patients treated with ASIs compared to placebo. Subgroup analysis showed that both Lorundrostat and Baxdrostat led to significant decreases in serum aldosterone, while no significant change was observed with Osilodrostat. These findings are consistent with the BrigHTN trial, where all three Baxdrostat doses reduced serum aldosterone levels after 12 weeks [23]. Similarly, Laffin et al. reported a significant reduction in serum aldosterone with Lorundrostat [22].

Among the ASIs, Osilodrostat did not demonstrate a significant reduction in serum aldosterone, likely due to its limited selectivity. CYP11B2, which encodes aldosterone synthase, shares over 93% sequence homology with CYP11B1, the enzyme responsible for cortisol synthesis [22, 30]. As reported by Calhoun et al., Osilodrostat inhibited both enzymes, resulting in off‐target effects; 20% of participants exhibited an inadequate cortisol response to ACTH stimulation [27, 38]. Additionally, the drug led to elevated levels of 11‐deoxycorticosterone, a potent mineralocorticoid receptor agonist [27]. In contrast, Lorundrostat, as demonstrated by Shimizu et al. and Hartmann et al., showed high selectivity for CYP11B2 over CYP11B1 (374:1 in preclinical assays), thereby minimising off‐target endocrine effects [31, 39]. Baxdrostat also yielded promising results in the BrigHTN trial, with a selectivity ratio of 100:1 and a comparable placebo‐subtracted blood pressure reduction in phase 2 dose‐ranging studies [23].

Fourth, regarding the risk of hyperkalemia, our analysis revealed a significantly higher incidence in patients treated with ASIs compared to placebo. This finding is consistent with reports by Freeman et al., Laffin et al. and Tuttle et al., where moderate hyperkalemia (serum potassium 5.6–5.9 mmol/L) occurred more frequently in patients receiving Lorundrostat, Baxdrostat and Vicadrostat [22, 23, 40]. Severe hyperkalemia (serum potassium ≥ 6.0 mmol/L), requiring dose adjustments or treatment interruption, remained rare—reported in 1.3%, 2.4% and 3.6% of patients on Vicadrostat, Baxdrostat and Lorundrostat, respectively, versus none in placebo groups [22, 23, 40]. These low rates may be partially explained by the exclusion of patients with high‐normal baseline serum potassium levels (4.8–5.0 mmol/L) and the relatively preserved kidney function in the Baxdrostat and Lorundrostat trials, as well as near‐normal renal function in the Vicadrostat trial by Tuttle et al. [22, 23, 40]. With regard to patients with impaired kidney function, the risk of hyperkalemia associated with ASI therapy is an important safety concern. In a Phase II trial of Vicadrostat by Tuttle et al., involving patients with CKD and albuminuria (eGFR 30–< 90 mL/min/1.73 m^2^), investigator‐reported hyperkalemia occurred in 10%, 15% and 18% of patients receiving 3 mg, 10 mg and 20 mg doses, compared to just 6% in the placebo group (*p* < 0.05.) importantly, most hyperkalemia events were mild and did not require intervention, yet they highlight a clear dose‐dependent safety signal [41]. These findings are consistent with the well‐documented hyperkalemia risk of RAAS inhibitors in CKD populations, where declining eGFR predicts elevated serum potassium and drives frequent treatment discontinuations [41].

Lastly, regarding the incidence of serious and non‐serious adverse events, our analysis showed comparable rates between the ASI and placebo groups, highlighting a favourable safety profile; particularly for first‐generation ASIs. This is likely due to the strict patient selection in early trials, which included individuals with lower cardiovascular risk and fewer hypertension‐related complications [42, 43]. These findings are consistent with prior ASI studies across varied populations, such as healthy volunteers, patients with uncontrolled or resistant HTN, and those with primary aldosteronism, where both surgical and medical treatments remain suboptimal [37, 44, 45]. This further supports the therapeutic potential of ASIs in HTN management.

### 4.1 Limitations

This systematic review and meta‐analysis has several limitations. First, six out of the eight included studies were Phase II trials with small sample sizes and short follow‐up durations (ranging from 8 to 12 weeks) which limits the ability to draw robust conclusions about the long‐term safety and efficacy of ASIs. Consequently, larger, well‐powered Phase III studies are warranted to validate these findings. Although the included RCTs were of high quality, their generalisability may be restricted due to variations in sample size, study design, patient characteristics, and the different types and doses of ASIs used.

Second, our analysis included multiple ASIs (Baxdrostat, Lorundrostat, Osilodrostat) administered at varying doses across different trials. This heterogeneity in drug type and dosage may have contributed to variability in treatment effects, limiting the ability to draw definitive conclusions regarding the efficacy and safety of individual agents.

Third, in our analysis, Osilodrostat was associated with a modest reduction in SBP but showed no significant effect on DBP. Additionally, no significant decrease in serum aldosterone levels was observed, despite its mechanism targeting steroidogenesis. This result is based on a single study in the subgroup and should therefore be interpreted cautiously. Lastly, publication bias was not formally assessed due to the small number of included studies (*n* = 8), which limits the reliability of funnel plots or statistical tests such as Egger's regression. As a result, the possibility of selective reporting or unpublished negative results cannot be excluded.

Despite these limitations, one of the strengths of our review is the use of subgroup analyses to explore treatment effect variability and better interpret heterogeneity across studies.

## 5 Conclusion

In conclusion, ASIs effectively reduce systolic blood pressure by selectively targeting aldosterone production, demonstrating significant clinical benefits in HTN management. The favourable safety profile observed, particularly among first‐generation ASIs, is supported by comparable rates of serious and non‐serious adverse events relative to placebo. While the increased incidence of hyperkalemia warrants careful patient selection and monitoring, this risk remains manageable within controlled trial settings. Overall, ASIs represent a promising therapeutic advancement for patients with HTN, especially those with resistant or difficult‐to‐treat forms, highlighting the need for further large‐scale and long‐term studies to fully elucidate their clinical utility and optimise treatment protocols.

## Author Contributions

Conceptualisation, data curation and project administration were carried out by J.S.G., S.S. and M.A.R., supervision was carried out by M.A., R.A. and M.S., formal analysis of data was carried out by Z.Z.C., N.B.P., and S.A., formal analysis, methodology and software were carried out by S.S., M.A.R., A.M., and M.A., writing the original draft was carried out by J.S.G., S.S., M.A.R., A.M., M.A., R.A., A.A., B.Y., and H.A., writing, reviewing and editing were carried out by M.A.W.K., A.A., A.A., and A.H., visualisation and validation were carried out by M.A., R.A., and M.S., S.A.

## Ethics Statement

The authors have nothing to report.

## Consent

The authors have nothing to report.

## Conflicts of Interest

The authors declare no conflicts of interest.

## Supporting information


**Data S1:** edm270094‐sup‐0001‐supinfo.docx.

## Data Availability

Data sharing is not applicable to this article as no new data were created or analyzed in this study.
