# Supplementary material for: Efficacy and Safety of Aldosterone Synthase Inhibitors in Hypertension: A Systematic Review and Meta‐Analysis
Source: Endocrinol Diabetes Metab. 2025 Sep 12;8(5):e70094. doi: 10.1002/edm2.70094 (PMC12432333; doi:10.1002/edm2.70094)

**Supplementary Materials**

**Contents:**

**Tables.**Table S1: Search strategy table.

Table S2: Studies Excluded from Citation Chasing with Corresponding Rationale

**Figures**.

Figure S1: Risk of Bias 2 traffic light plot.

**Figure S2: Leave-one-out sensitivity analysis of SBP reduction**

**Figure S3: Leave-one-out sensitivity analysis of DBP reduction**

**Figure S4: Leave-one-out sensitivity analysis of serious adverse events excluding the HALO trial**

**Figure S5: Leave-one-out sensitivity analysis of non-serious adverse events**

**Figure S6: Leave-one-out sensitivity analysis of non-serious adverse events excluding the HALO trial**

**Figure S7: Leave-one-out sensitivity analysis of serum aldosterone levels**

**Figure S8: Leave-one-out sensitivity analysis of serum aldosterone levels excluding the HALO trial**

**Figure S9: Leave-one-out sensitivity analysis of hyperkalemia risk**

**Table S1: Search Strategy Table**

| **Database** | **Search Strategy** | **Results** |
| --- | --- | --- |
| **PubMed** | (("aldosterone synthase inhibitor" OR lorundrostat OR osilodrostat OR baxdrostat) AND ("hypertension" OR "primary hypertension" OR "uncontrolled hypertension" OR "treatment-resistant hypertension") | 86 |
| **Google Scholar** | (("aldosterone synthase inhibitor" OR lorundrostat OR osilodrostat OR baxdrostat) AND ("hypertension" OR "primary hypertension" OR "uncontrolled hypertension" OR "treatment-resistant hypertension") | 1,340 |
| **Cochrane Library** | (("aldosterone synthase inhibitor" OR lorundrostat OR osilodrostat OR baxdrostat) AND ("hypertension" OR "primary hypertension" OR "uncontrolled hypertension" OR "treatment-resistant hypertension") | 21 |

**Table S2: Studies Excluded from Citation Chasing with Corresponding Rationale**

| **Author Name** | **Year** | **Title** | **Journal** | **Reason for exclusion** |
| --- | --- | --- | --- | --- |
| Dogra et al. | 2023 | Baxdrostat: A Novel Aldosterone Synthase Inhibitor for Treatment Resistant Hypertension. | *Current*  *Problems in Cardiology* | Review |
| Olivas FJ et al. | 2024 | Diuretics use in the management of hypertension | *Hipertens Riesgo Vasc.* | Review |
| Jansen et al. | 2009 | Aldosterone synthase inhibitors: Pharmacological and clinical aspects. | *Curr Opin Investig Drugs* | Review |
| Cerny et al. | 2013 | Progress towards clinically useful aldosterone synthase inhibitors. | *Curr Top Med Chem*. | Review |
| Mulatero et al. | 2023 | CYP11B2 inhibitor dexfadrostat phosphate suppresses the aldosterone-to-renin ratio, an indicator of sodium retention, in healthy volunteers. | *Br J Clin Pharmacol.* | Review |
| Azizi et al. | 2013 | Aldosterone synthase inhibition in humans. | *Nephrology Dialysis Transplantation* | Review |
| Irfan et al. | 2024 | Hypertension and Lorundrostat: Key Discoveries From the TARGET-HTN Trial. | *Current Problems in Cardiology* | Review |
| Dey et al. | 2023 | Baxdrostat: An Aldosterone Synthase Inhibitor for the Treatment of Systemic Hypertension. | *Cardiol Rer.* | Review |
| Awosika et al. | 2023 | Aldosterone Synthase Inhibitors and Dietary Interventions: A Combined Novel Approach for Prevention and Treatment of Cardiovascular  Disease. | *Cureus* | Review |
| Groseljet al. | 2023 | Osilodrostat for Cushing Disease and Its Role in Pediatrics. | *Hormone Research in Paediatrics* | Review |
| Martins VM et al. | 2019 | Efficacy of chlorthalidone and hydrochlorothiazide in combination with amiloride in multiple doses on blood pressure in patients with primary hypertension: a protocol for a factorial randomized controlled trial. | *Trials* | Trial Protocol |
| Musini VM et al. | 2016 | Blood pressure-lowering efficacy of monotherapy with thiazide diuretics for primary hypertension. | *Cochrane Database Syst Rev.* | Meta-analysis |
| Chen et al. | 2018 | First-line drugs inhibiting the renin angiotensin system versus other first-line antihypertensive drug classes for hypertension | *Cochrane Database Syst Rev.* | Meta-analysis |
| McCann et al. | 2025 | Case-crossover assessment of the modifying effects of home medication use on acute kidney-related morbidity due to elevated ambient heat exposure in Atlanta, GA, from 2013 to 2019 | *BMJ Public Health.* | Retrospective study |
| Huang et al. | 2024 | Genome-Wide Methylation Analysis Reveals a KCNK3-Prominent Causal Cascade on Hypertension | *Circulation research.* | Retrospective study |
| Newby et al. | 2024 | The relationship between isolated hypertension with brain volumes in UK Biobank | *Brain and Behaviour* | Retrospective study |
| Ruopp et al. | 2022 | Diagnosis and Treatment of Pulmonary Arterial Hypertension: A Review | *JAMA* | Review |
| Mandras | 2021 | Combination Therapy in Pulmonary Arterial Hypertension-Targeting the Nitric Oxide and Prostacyclin Pathways | *J Cardiovasc Pharmacol Ther.* | Review |
| Monticone et al. | 2018 | Cardiovascular events and target organ damage in primary aldosteronism compared with essential hypertension: a systematic review and meta-analysis | *Lancet Diabetes Endocrinol.* | Meta-analysis |
| Saiz et al. | 2022 | Blood pressure targets for the treatment of people with hypertension and cardiovascular disease | *Cochrane Database Syst Rev.* | Review |
| Falk et al. | 2024 | Higher blood pressure targets for hypertension in older adults | *Cochrane Database Syst Rev.* | Review |

**Figure S1: Risk of Bias 2 traffic light plot.**

**
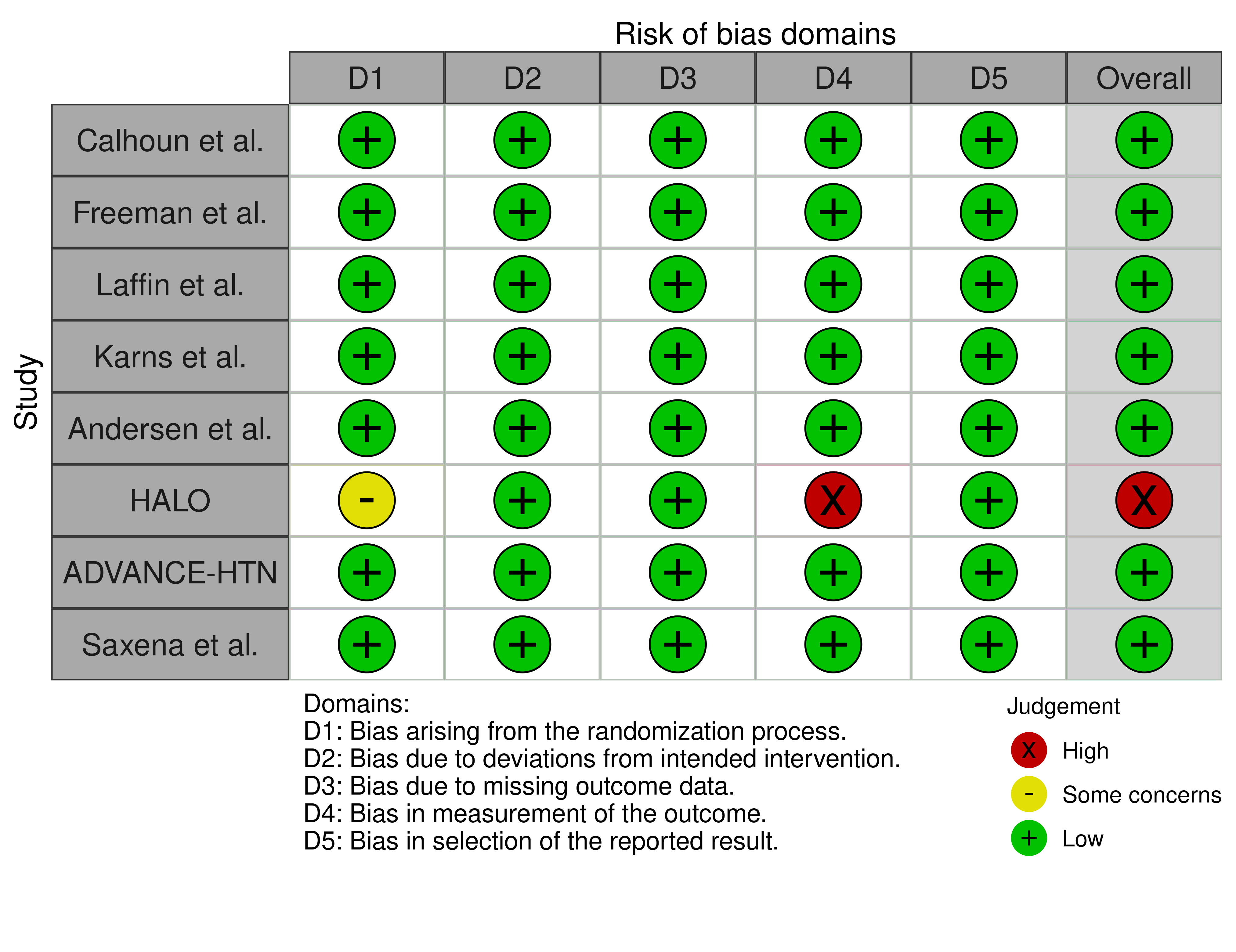
**

**Figure S2: Leave-one-out sensitivity analysis of SBP reduction**


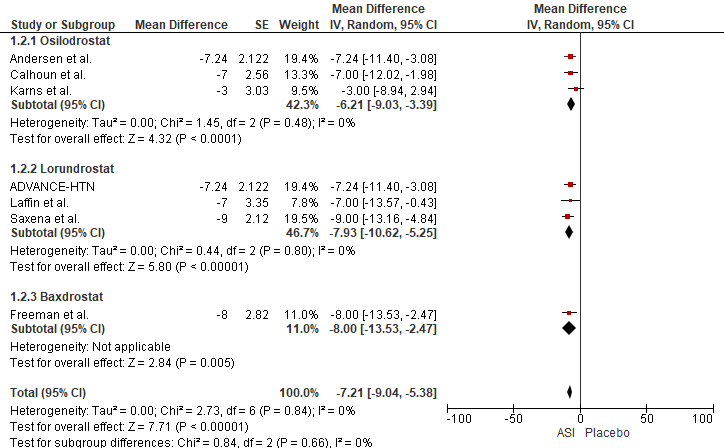


**Figure S3: Leave-one-out sensitivity analysis of DBP reduction**


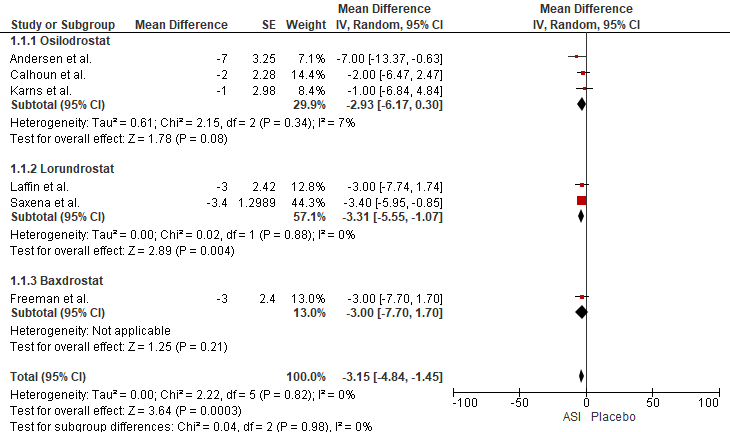


**Figure S4: Leave-one-out sensitivity analysis of serious adverse events excluding the HALO trial**

**
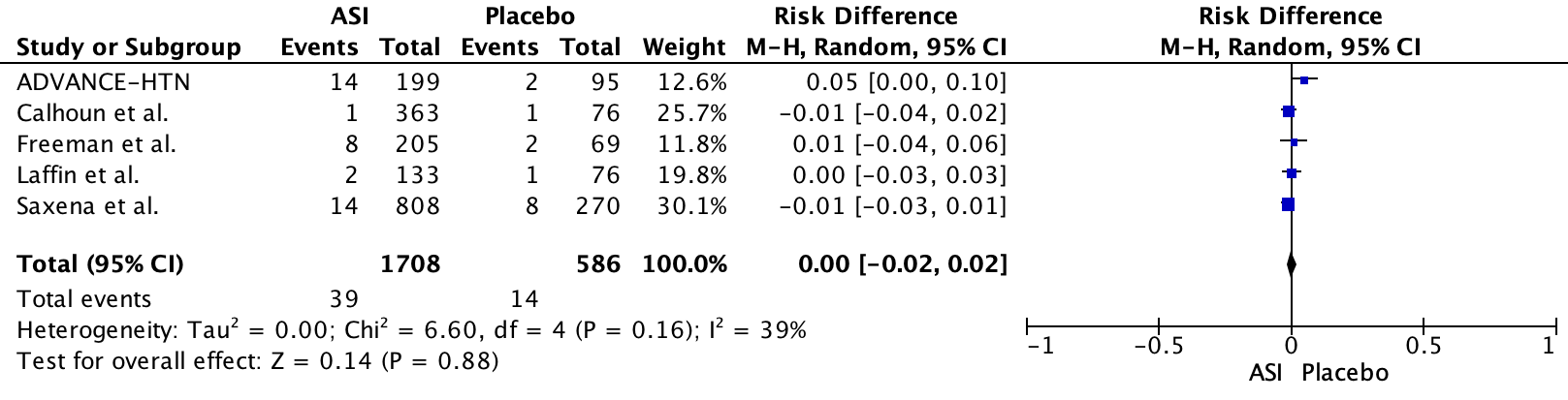
**

**Figure S5: Leave-one-out sensitivity analysis of non-serious adverse events**


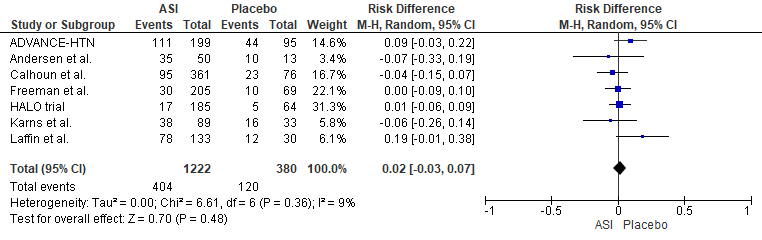


**Figure S6: Leave-one-out sensitivity analysis of non-serious adverse events excluding the HALO trial**

**
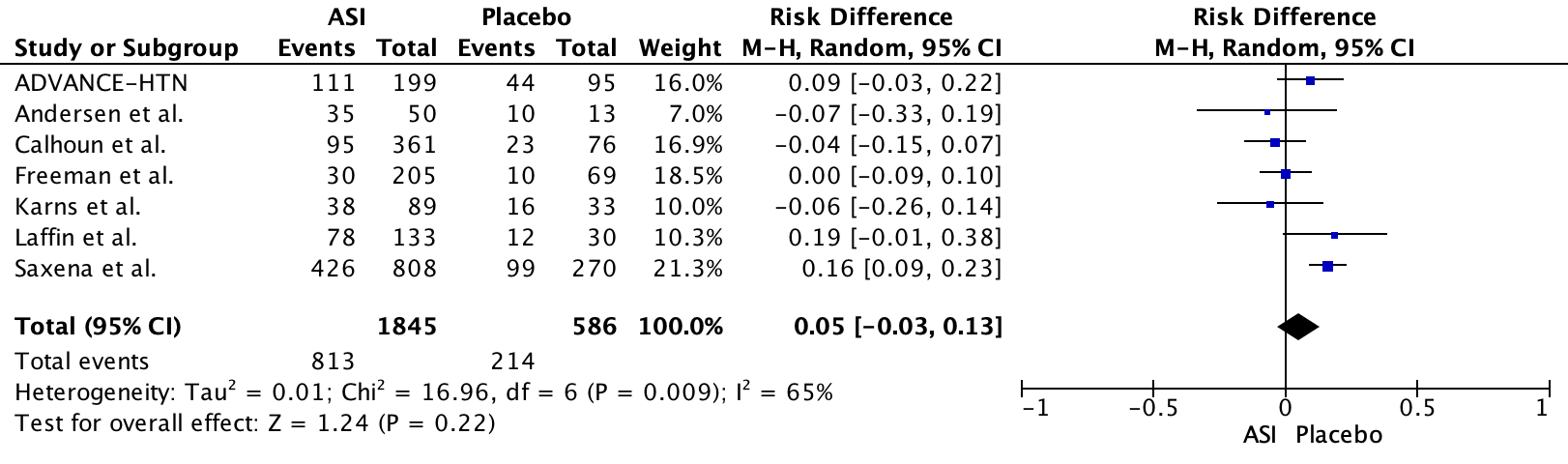
**

**Figure S7: Leave-one-out sensitivity analysis of serum aldosterone levels**


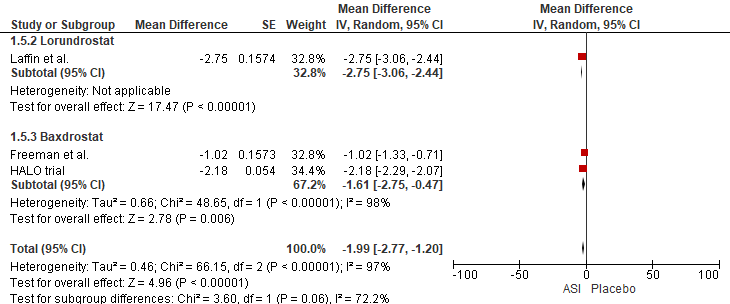


**Figure S8: Leave-one-out sensitivity analysis of serum aldosterone levels excluding the HALO trial**

**
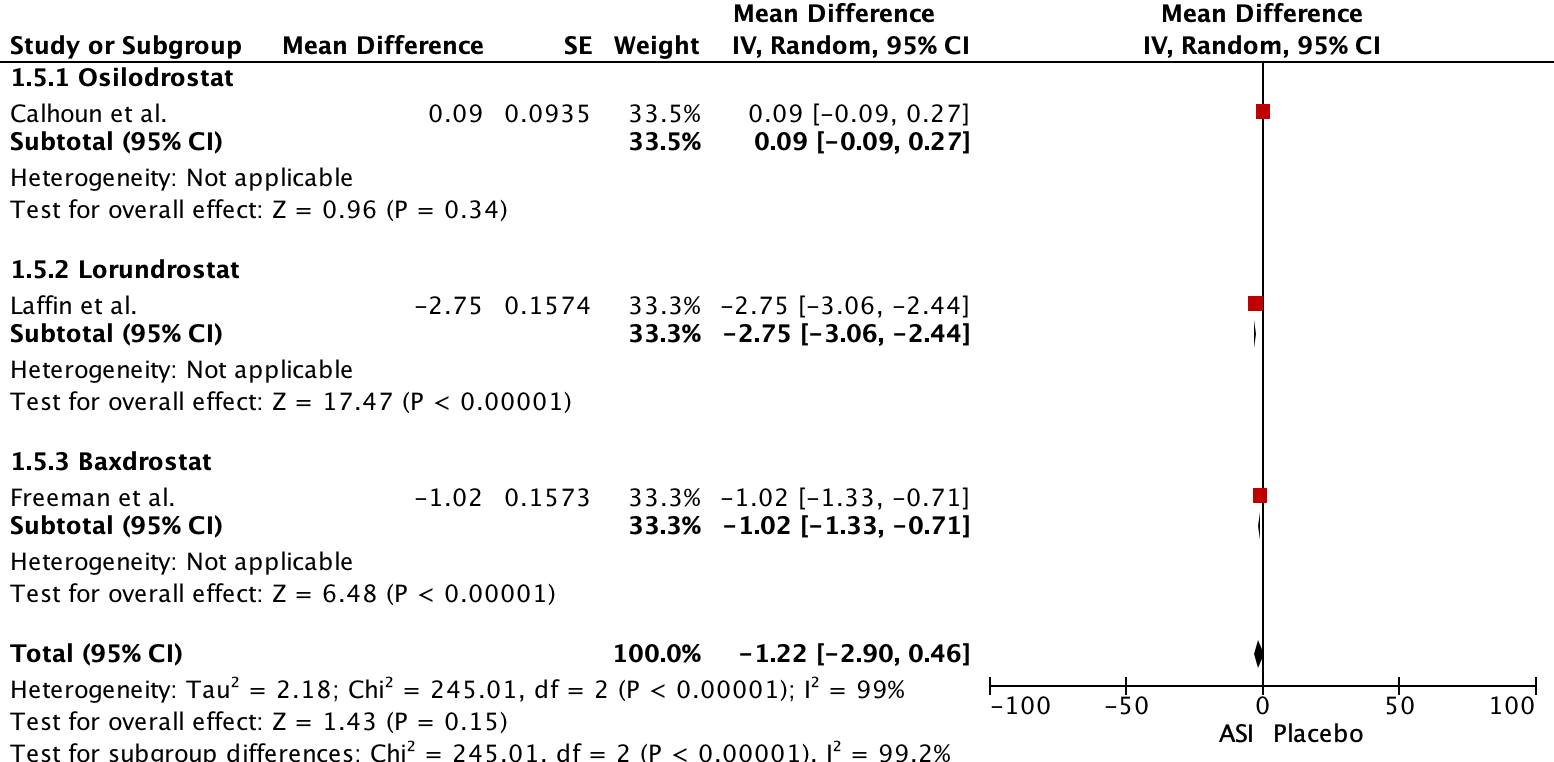
**

**Figure S9: Leave-one-out sensitivity analysis of hyperkalemia risk**


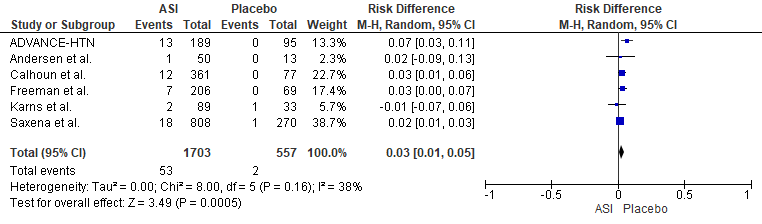

Supplement: Supplementary file 1 — Data S1: edm270094‐sup‐0001‐supinfo.docx. [file EDM2-8-e70094-s001.docx]
